# Cervical Spine Degeneration in Rugby Players: Position-Specific Differences in Radiographic and Clinical Outcomes Among 64 Brazilian Athletes

**DOI:** 10.3390/jfmk11010043

**Published:** 2026-01-20

**Authors:** Matheus Neves Castanheira, Yoshinobu Nagasse, Michel Kanas, Nelson Astur, Délio Eulálio Martins Filho, Felipe Neves Simões Monteiro, Marcelo Wajchenberg

**Affiliations:** 1 Department of Orthopedics and Traumatology, Hospital Israelita Albert Einstein, Sao Paulo 05652-900, SP, Brazil; michelkanas@gmail.com (M.K.); nelsonan@yahoo.com (N.A.); deliomartins.br@gmail.com (D.E.M.F.); drfelipenevesmonteiro@gmail.com (F.N.S.M.); marcelow@einstein.br (M.W.); 2 Instituto Cohen de Ortopedia, Reabilitação e Medicina do Esporte, Sao Paulo 05652-900, SP, Brazil; 3 Deparment of Orthopedics and Traumatology, Federal University of Sao Paulo (UNIFESP), Sao Paulo 04039-002, SP, Brazil; ynagasse@yahoo.com.br

**Keywords:** rugby, cervical spine, degeneration, sagittal alignment, athletes

## Abstract

**Background:** Rugby exposes athletes to high mechanical loads, especially during scrums and tackles, potentially predisposing players to early cervical spine degeneration. This study evaluated the prevalence of degenerative changes in the cervical spine and sagittal alignment alterations in Brazilian rugby athletes, with secondary analyses comparing forwards and backs and examining associations between alignment parameters and pain and disability. **Methods:** Sixty-four professional rugby athletes underwent cervical spine radiography, and the images were analyzed for degenerative findings and sagittal parameters (cervical lordosis, T1 slope, cervical sagittal vertical axis, and T1–CL mismatch). Pain and disability were assessed using the Visual Analogue Scale (VAS) and Neck Disability Index (NDI). Comparative analyses included Student’s *t*-test and Fisher’s exact test, while additional exploratory analyses were performed using correlation and multiple linear regression models. **Results:** Cervical degeneration was present in 20.3% of players. Forwards reported significantly greater pain than backs (VAS: 1.64 ± 1.58 vs. 0.76 ± 0.93; *p* = 0.007). Deviations in cervical lordosis (>2 SD from normative values) were associated with higher VAS scores (*p* = 0.024). No significant associations were found between T1 slope or cervical sagittal vertical axis and pain or disability. **Conclusions:** Forwards demonstrated greater symptom burden and a higher prevalence of cervical degenerative changes, suggesting that positional demands may contribute to early cervical spine alterations. These findings highlight the need for targeted preventive strategies and support future longitudinal investigations to clarify the progression and clinical relevance of cervical misalignment in collision-sport athletes.

## 1. Introduction

Rugby is a high-intensity sport that exposes players to significant mechanical forces, especially during scrums, tackles, and rucks [1,2]. Players are divided into two broad positional groups: forwards and backs. Forwards (props, hookers, and locks) endure repetitive axial compression during scrums (up to 1500–2000 N per engagement) [3,4], while backs (halves and wings) experience high-velocity tackles and cervical hyperextension [5]. These biomechanical disparities may underlie positional differences in the risk of degeneration. Radiographic findings suggestive of degeneration include vertebral endplate sclerosis, reduced disc height, and osteophyte formation [6,7].

The pathophysiology of disc degeneration in the context of repetitive trauma is described well by the Kirkaldy–Willis model [8], which proposes that annular injuries initiate a progressive degenerative cascade. Although the Kirkaldy–Willis degeneration model was originally developed to describe the progressive cascade of lumbar disc injury, subsequent research has demonstrated that its principles are applicable to the cervical spine. Several studies have shown that repetitive microtrauma, segmental instability, and subsequent structural stiffening follow a similar degenerative sequence in cervical motion segments, supporting the relevance of this framework for understanding cervical pathology in collision-sport athletes [9]. More recent evidence has reinforced these concepts: population-based MRI studies report findings of cervical degeneration in up to 25–35% of asymptomatic individuals under 30 years of age, with prevalence increasing significantly in athletes exposed to high-impact or repetitive axial-loading environments [10]. Rugby players in particular show higher rates of disc height loss, osteophyte formation, and Modic changes compared with age-matched controls, highlighting the growing epidemiological impact and the need for sport-specific investigations, such as the present study [6,7].

Over the last decade, rugby has become more popular in Brazil, creating a solid base of athletes and increasing exposure to collision-related cervical loads [11]. The sport’s technical complexity and physical demands require biomechanical resilience, as scrums, tackles, and rucks impose substantial forces on the cervical spine, particularly among forwards, while high-speed impacts and sudden directional changes also affect backs [3,4].

Despite growing evidence of cervical degeneration in collision-sport athletes, most available studies focus on retired players, isolated imaging findings, or non-position-specific analyses, with limited integration of radiographic alignment parameters and clinical outcomes [3,4]. Moreover, data examining position-specific radiographic–clinical correlations in active professional rugby players remain scarce, particularly in Brazilian and Latin American cohorts.

Therefore, this study was performed to address this gap by evaluating cervical degenerative changes, sagittal alignment, and their clinical associations in professional Brazilian rugby athletes, with a specific focus on positional differences between forwards and backs.

## 2. Objective

The primary objective of this study was to determine the prevalence of degenerative changes in the cervical spine and sagittal alignment alterations in professional Brazilian rugby athletes.

The secondary objectives were [1] to compare radiographic and clinical parameters between forwards and backs, [2] to analyze whether deviations in cervical sagittal alignment were associated with self-reported pain (VAS) and functional status (NDI), and [3] to perform exploratory analyses comparing radiographic parameters with published normative values and examine the influence of demographic variables, including age and sex, on clinical outcomes.

## 3. Materials and Methods

### 3.1. Study Design and Participants

This cross-sectional study included 64 professional athletes from the Brazilian Rugby Confederation. The inclusion criteria were regular rugby practice (≥3 training sessions per week) and an age between 18 and 40 years. Although all participants are professional athletes registered with the Brazilian Rugby Confederation, training volume can vary during competitive and off-season periods, and some athletes may temporarily reduce their activity due to scheduling conflicts or minor injuries. Therefore, a criterion of “regular practice ≥ 3 sessions per week” was used to ensure a minimum and homogeneous level of exposure to rugby-related mechanical loads, avoiding the inclusion of players who were inactive or in a reduced training program at the time of recruitment. The non-inclusion criterion was a lack of availability to undergo cervical radiographic evaluation. The exclusion criteria were a history of cervical spine surgery, previously diagnosed traumatic cervical injuries unrelated to rugby (e.g., car accidents, high-energy trauma), and known congenital cervical spine abnormalities, such as Klippel–Feil syndrome or congenital fusion anomalies. These criteria were applied to avoid potential confounding factors that could influence radiographic degeneration or alignment independently of rugby-related mechanical exposure. Participants were recruited through the Brazilian Rugby Confederation. The study was presented to medical and coaching staff, and all eligible players from the senior squads were invited to participate during routine team activities and medical screening sessions. Athletes who agreed to participate signed an informed consent form and were then scheduled for cervical spine radiographs and completion of the questionnaires. All participants provided informed consent, and this study received approval from the Research Ethics Committee of Hospital Israelita Albert Einstein (CAAE 26186719.0.0000.5505). This study followed the STROBE guidelines for observational research [12].

### 3.2. Data Collection

Participants completed structured questionnaires documenting demographic information, years of rugby experience, playing position, and neurological symptoms. Pain intensity was assessed using the Visual Analogue Scale (VAS), and disability was assessed using the Neck Disability Index (NDI) and expressed as a percentage score (hereafter referred to as IIRP) that reflects patient-reported disability in daily activities, including self-care, work, and recreational tasks. Both scales demonstrate strong validity and reliability: the VAS shows excellent test–retest reliability and sensitivity for detecting changes in musculoskeletal pain, while the NDI is a well-validated instrument with robust psychometric properties for assessing cervical-related disability [13].

## 4. Radiographic Acquisition and Assessment

**Figure 1:**
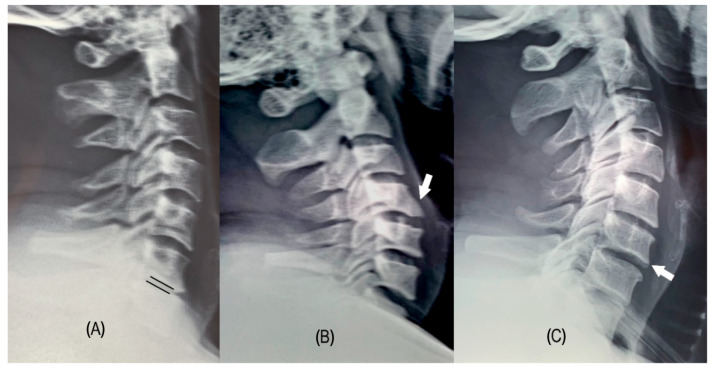
Examples of cervical degeneration: (**A**) A lateral cervical radiograph showing decreased disc space between parallel black lines, compared with adjacent levels. (**B**) A lateral cervical radiograph with an arrow indicating an anterior osteophyte at the C4 vertebral body. (**C**) A lateral cervical radiograph with an arrow highlighting sclerosis of the inferior endplate of C6. Source: Personal archive.

All participants underwent standardized anteroposterior and lateral cervical spine radiography. Degenerative changes were defined as loss of disc height > 50% compared with adjacent levels and/or the presence of vertebral endplate sclerosis, osteophytes, or spondylolisthesis (Figure 1). Sagittal parameters—including cervical lordosis (C2–C7 Cobb angle), T1 slope (T1S), cervical sagittal vertical axis (cSVA), and T1–CL mismatch—were measured using Surgimap^®^ (version 2.3.2.1; Nemaris Inc., New York, NY, USA).

**Figure 2:**
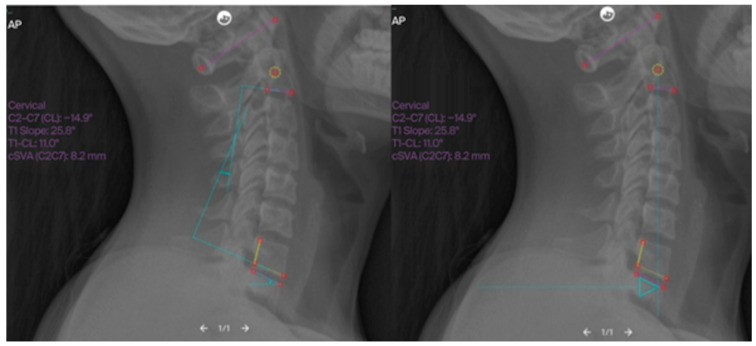
Examples of CL, T1S, and cSVA measurements using Surgimap^®^. (**Left**): Lateral cervical radiograph with measurements of cervical lordosis and T1 slope. (**Right**): Lateral cervical radiograph with measurement of cervical sagittal vertical axis. Source: Surgimap^®^ software, version 2.3.2.1 (Nemaris Inc., New York, NY, USA).

**Figure 3:**
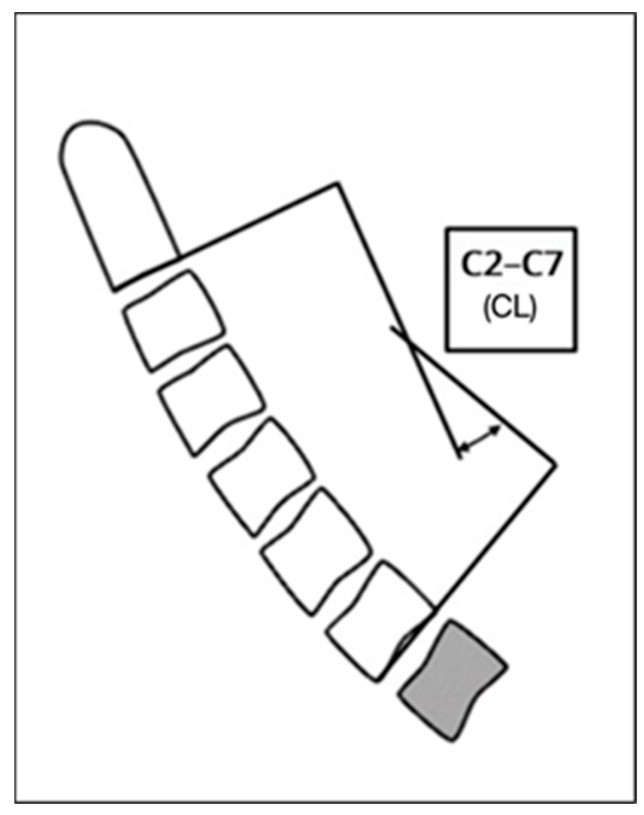
The Cobb method for measuring cervical lordosis. A schematic of the cervical spine. The shaded object represents the T1 vertebra. Source: Adapted from Surgimap^®^ (version 2.3.2.1; Nemaris Inc., New York, NY, USA).

**Figure 4:**
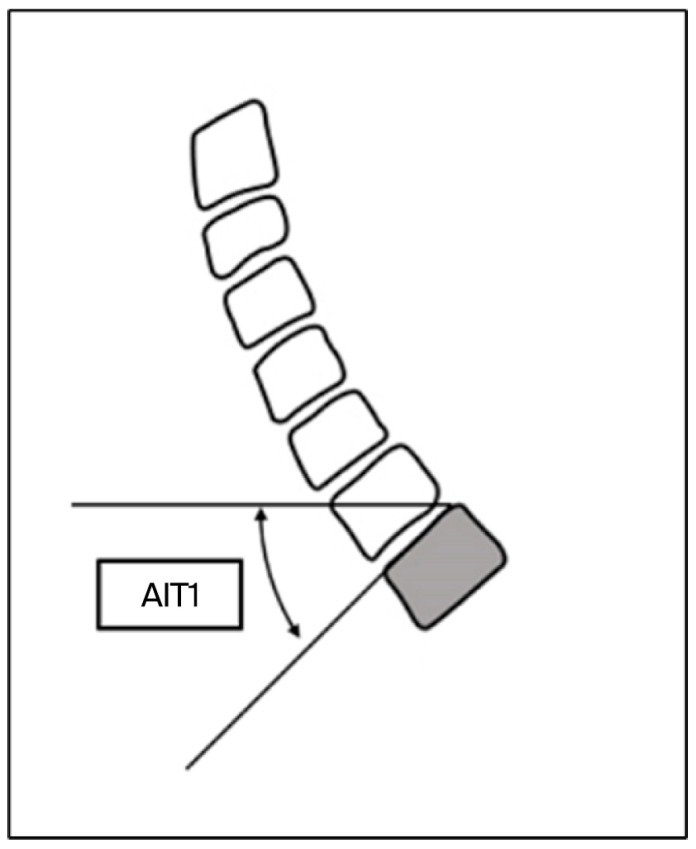
The method for measuring the T1 slope. The shaded object represents T1. Source: Adapted from Surgimap^®^ (version 2.3.2.1; Nemaris Inc., New York, NY, USA).

**Figure 5:**
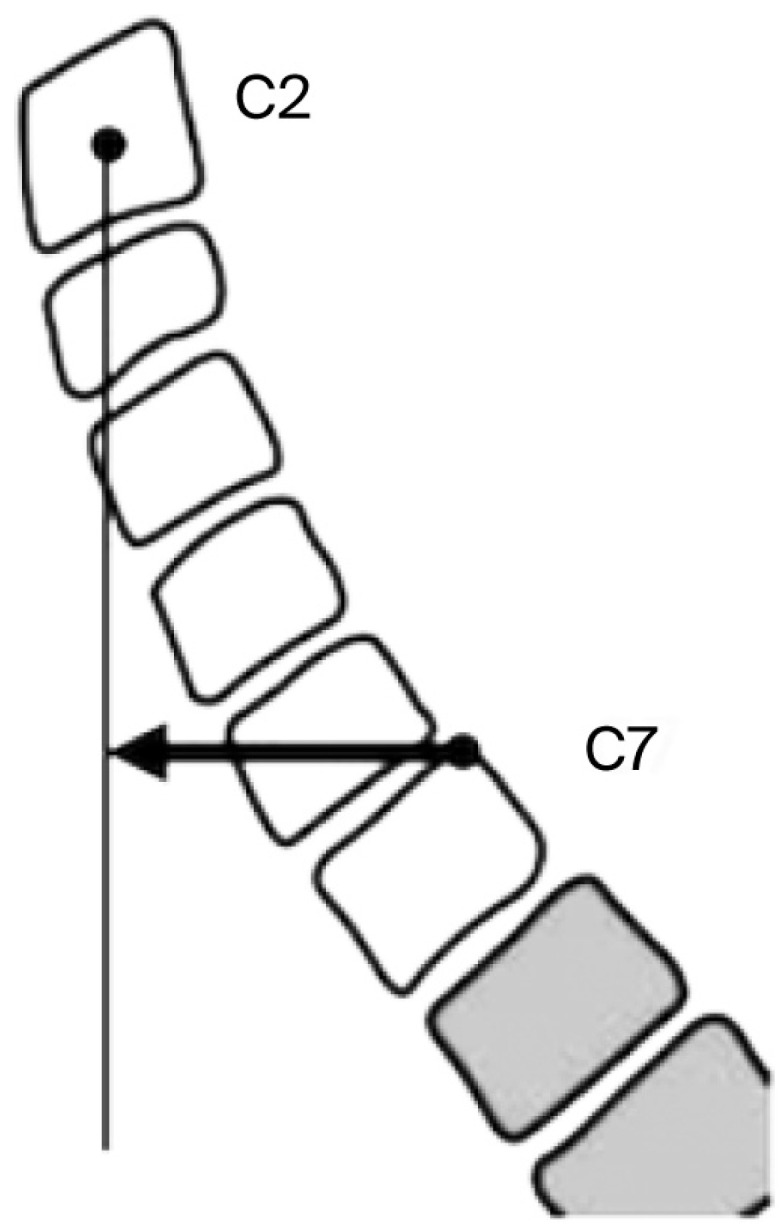
The method for measuring the cSVA, with a vertical line from the centroid of C2. The length of the arrow represents the cSVA. Source: Adapted from Surgimap^®^ (version 2.3.2.1; Nemaris Inc., New York, NY, USA).

**Figure 6:**
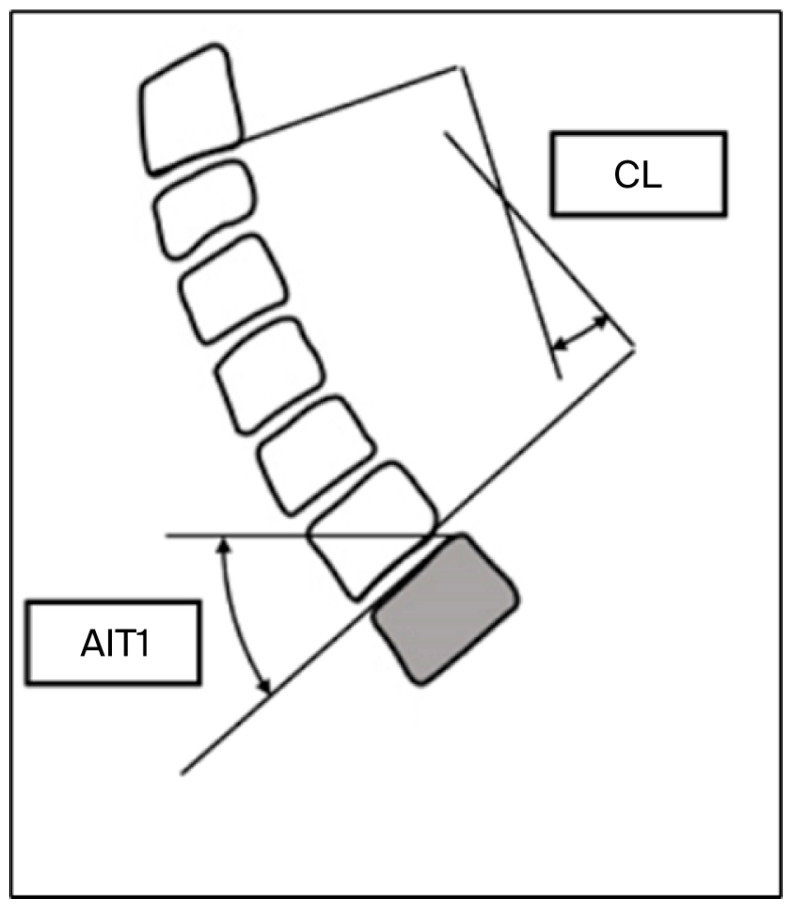
The method for measuring T1–CL mismatch. Source: Adapted from Surgimap^®^ (version 2.3.2.1; Nemaris Inc., New York, NY, USA).

Cervical lordosis was measured between the inferior endplates of C2 and C7 using the Cobb method, as illustrated in Figure 2 and Figure 3, which demonstrate the identification of anatomical landmarks and angle construction. The T1 slope was defined as the angle between the superior endplate of T1 and the horizontal line, as shown in Figure 4. The cervical sagittal vertical axis (cSVA) was measured as the horizontal distance between the centroid of C2 and a vertical line drawn from the posterior–superior corner of C7, as illustrated in Figure 5. The T1–CL mismatch was calculated as the numerical difference between the T1 slope and cervical lordosis, as shown in Figure 6.

These figures illustrate the radiographic measurement techniques applied in this study and clarify landmark selection and measurement orientation and do not present primary outcome data.

Manual identification of bony landmarks (e.g., vertebral endplates and the centroid of C2) was first performed by a single evaluator, after which the system automatically calculated the corresponding angular and linear measurements. Surgimap^®^ has demonstrated excellent intra- and interobserver reliability for cervical sagittal measurements, with intraclass correlation coefficients (ICCs) typically reported between 0.89 and 0.98 in validation studies. This software is widely used in spinal research and has been validated for the measurement of cervical lordosis, T1 slope, and cervical sagittal vertical axis, supporting its methodological robustness for radiographic assessment [14,15].

All radiographs were assessed by a single experienced orthopedic spine surgeon, who performed both the radiographic inspection and all angular and linear measurements. To minimize potential bias, the observer was blinded to the participants’ clinical data, including their VAS and NDI scores, neurological symptoms, and playing position.

Radiographic Measurements (Surgimap^®^ (version 2.3.2.1; Nemaris Inc., New York, NY, USA) [14]:

- C2–C7 Cobb Angle (Cervical Lordosis, CL)—the angle traced between the posterior–inferior borders of C2 and C7. Values above 36.2° or below −6.2° were considered altered, corresponding to >2 SD from population means [15].
- T1 Slope (T1S)—the angle between the superior endplate of T1 and the horizontal. Values below 9.6° or above 41.2° were considered altered (>2 SD) [15].
- Cervical Sagittal Vertical Axis (cSVA)—the horizontal distance between the centroid of C2 and a line perpendicular to the posterior edge of C7. Values below −5.1 mm or above 41.3 mm were considered altered (>2 SD) [15].
- T1–CL Mismatch (ΔT1–CL)—the T1 slope minus cervical lordosis. Values > 15° were considered altered [15].

### Statistical Analysis

Descriptive statistics were used to summarize the demographic, clinical, and radiographic characteristics of the sample. Altered radiographic parameters were defined as values beyond ±2 standard deviations from normative data in the literature. Student’s independent samples *t*-test was used for comparisons between forwards and backs, while a one-sample Student’s *t*-test was applied when comparing radiographic parameters from our cohort with published normative values. Fisher’s exact test was used for categorical variables. Pearson’s correlation coefficient (r) was used to assess the relationship between age and VAS and NDI scores, and both r-values and the corresponding *p*-values were reported. Multiple linear regression models were constructed for the VAS and NDI scores, incorporating variables with *p* < 0.10 in the bivariate analyses. All analyses were conducted using IBM SPSS v22.0, adopting a significance level of 5%.

For the analyses presented in Table 1 the athletes were categorized according to the presence or absence of altered sagittal alignment parameters, defined as values outside ±2 standard deviations from published normative data. Between-group comparisons of normal versus altered parameters were performed using Fisher’s exact test for categorical variables and Student’s independent samples *t*-test for continuous variables. In cases where no events were observed in one of the comparison groups, Fisher’s exact test was used, and non-significant *p*-values reflect the absence of statistically detectable associations rather than evidence of equivalence.

## 5. Results

### 5.1. Participant Characteristics

The mean age was 24.1 ± 3.4 years, with equal sex distribution. Most athletes (81.3%) reported more than three years of rugby practice, and 60.9% played as forwards. Degenerative cervical changes were identified in 20.3% of participants, characterized by osteophytes, disc height loss, and vertebral endplate sclerosis (Table 2).

#### 5.1.1. Radiographic Parameters and Comparison with Normative Values

The mean cervical lordosis (CL) was 19.8° ± 14.1, the mean T1 slope (T1S) was 28.1° ± 8.1, and the mean cervical sagittal vertical axis (cSVA) was 12.7 ± 10.2 mm. The mean T1–CL mismatch was 8.3° ± 10.4 (Table 3).

When compared with published normative values, the mean cSVA was significantly lower in the study cohort (*p* = 0.004), whereas CL and T1S did not differ significantly from the reference values (Table 1).

#### 5.1.2. Clinical Outcomes (VAS and NDI)

The overall mean VAS score was 1.3 ± 1.42. Forwards reported significantly higher pain scores than backs (1.64 ± 1.58 vs. 0.76 ± 0.93; *p* = 0.007) (Table 4). Although statistically significant, the mean VAS values remained below the threshold of clinical relevance (VAS ≥ 4).

The mean Neck Disability Index (NDI) score was 4.4% ± 5.1, corresponding to the “no disability” category. Male athletes presented significantly lower NDI scores compared with females (*p* = 0.039) (Table 5).

#### 5.1.3. Associations Between Alignment, Position, and Clinical Outcomes

Altered cervical lordosis values (>2 SD from normative values) were associated with higher VAS scores (*p* = 0.024) and higher NDI scores (*p* = 0.027) (Table 4 and Table 5). No significant associations were identified between T1 slope or cSVA and pain or disability outcomes.

A history of neurological symptoms (including paresthesia and stingers) was reported by 29.7% of participants and was not significantly associated with cervical degeneration or sagittal alignment parameters.

In multiple linear regression analysis, playing position (forward) showed a trend of association with higher VAS scores (*p* = 0.057), while sex and altered cervical lordosis remained independently associated with NDI outcomes (Table 5).

## 6. Discussion

### 6.1. Prevalence of Cervical Degenerative Changes

This study identified a 20.3% prevalence of degenerative cervical changes among professional Brazilian rugby players (Table 2), a finding consistent with previous reports in collision sports [10,16,17] and higher than the rate described in age-matched general populations, which ranges from approximately 12% to 17% [16,17]. This finding directly addresses the primary objective of the study, demonstrating that cervical degeneration is relatively common even in young, active professional rugby players.

The presence of disc height reduction, osteophyte formation, and vertebral endplate sclerosis reinforces the role of repetitive axial loading and cumulative microtrauma, as described in biomechanical and degenerative cascade models [4,5,6]. These observations align with prior investigations in rugby and other collision sports, supporting the hypothesis that sport-specific mechanical demands contribute to early structural changes [2,10].

### 6.2. Positional Biomechanics and Pain Outcomes

In accordance with the secondary objective of comparing forwards and backs, forwards reported significantly higher pain scores than backs (VAS: 1.64 ± 1.58 vs. 0.76 ± 0.93; *p* = 0.007; Table 4). This result is biomechanically plausible, given the greater exposure of forwards to repetitive axial compression and shear forces during scrums and rucks [3,18].

Although forwards did not demonstrate a significantly higher prevalence of radiographic degeneration compared with backs, the greater symptom burden observed in this group suggests that pain may occur independent of overt structural degeneration. This observation reinforces the concept that cervical pain in collision-sport athletes may reflect cumulative mechanical stress and neuromuscular factors rather than radiographic degeneration alone [16,17].

### 6.3. Sagittal Alignment and Clinical Correlates

Deviation in cervical lordosis beyond ±2 standard deviations from normative values was associated with higher VAS scores (Table 3) and remained independently associated with higher NDI scores in multivariate analysis (Table 5). This finding addresses the secondary objective of evaluating the relationship between sagittal alignment and clinical outcomes and is consistent with prior studies linking cervical misalignment to worse pain and disability metrics [15,19].

In contrast, no significant associations were identified between T1 slope or cervical sagittal vertical axis and pain or disability outcomes. These findings may be partly explained by the limited sensitivity of plain radiographs in detecting early functional or disc-related pathology and may also reflect the cross-sectional design and sample size of the present study [19].

### 6.4. Degeneration, Symptoms, and Multifactorial Pain

Despite the identification of cervical degenerative changes in approximately one-fifth of the cohort, no strong association was observed between radiographic degeneration and pain or disability scores. This observation is in line with previous reports demonstrating weak or inconsistent correlations between imaging findings and clinical symptoms, particularly in young and athletic populations [16,17].

These results support the concept that cervical pain in rugby players is multifactorial and likely influenced by neuromuscular control, training load, acute microtrauma, and biomechanical exposure rather than structural degeneration alone [2,19].

### 6.5. Sex-Related Differences in Disability

Male athletes demonstrated significantly lower NDI scores compared with female athletes (Table 5). Although sex-based analyses were exploratory and not a primary objective of the study, this finding may be partially explained by greater cervical muscle mass and neck strength in male athletes, which could confer improved stabilization and protection against repetitive mechanical stress [20].

Given the exploratory nature of this analysis and the balanced sex distribution in the cohort, these findings should be interpreted with caution but warrant further investigation in larger, sex-stratified samples.

In summary, this study provides an integrated radiographic and clinical assessment of the cervical spine in professional Brazilian rugby players, highlighting position-specific differences in pain and the clinical relevance of cervical sagittal alignment. While radiographic degenerative changes were relatively frequent, symptom burden appeared to be more closely related to biomechanical exposure and alignment alterations than to degeneration alone. These findings reinforce the importance of considering positional demands and sagittal balance when interpreting cervical symptoms in collision-sport athletes and provide a framework for future longitudinal and interventional investigations.

## 7. Limitations

This study has several limitations that should be acknowledged. First, the relatively small sample size may have limited the statistical power to detect weaker associations between radiographic parameters and clinical outcomes. Second, cervical spine assessment was based exclusively on plain radiographs, which may result in an underestimation of early degenerative disc changes or soft tissue alterations that are better characterized with advanced imaging modalities. Third, the absence of a non-athlete control group limits direct comparisons with the general population. Finally, participant recruitment was partially affected by restrictions imposed during the SARS-CoV-2 pandemic, which may have influenced sample size and imaging availability.

Future studies should address these limitations through longitudinal designs, larger multicenter cohorts, and the inclusion of appropriate control groups. The use of magnetic resonance imaging (MRI) may provide greater sensitivity for detecting early degenerative changes, while electromyography (EMG) could help elucidate neuromuscular activation patterns and neuromuscular control associated with cervical loading. In addition, interventional studies evaluating targeted neck-strengthening programs, scrum-technique optimization, and neuromuscular conditioning may help identify effective preventive strategies to reduce cervical pain and cumulative cervical overload in high-exposure playing positions.

## 8. Conclusions

In this cohort of professional Brazilian rugby players, forwards demonstrated a greater symptom burden and a relevant prevalence of findings indicating cervical degeneration. Alterations in cervical lordosis were associated with worse pain and disability outcomes, whereas other sagittal alignment parameters showed no consistent relationship with clinical measures.

Although the cross-sectional design and sample size do not allow causal inferences or definitive conclusions regarding position-specific degenerative risk, the observed patterns warrant closer clinical monitoring, particularly among forwards. These findings emphasize the importance of considering positional biomechanical demands and sagittal alignment when evaluating cervical symptoms in collision-sport athletes and support the need for longitudinal studies incorporating advanced imaging and preventive strategies.

## Figures and Tables

**Table 1:** Comparison of cervical radiographic parameters in this study’s cohort and published normative values.

Variable	Rugby Players	Literature [14]	*p*
	(N = 64)	(N = 44)	
CL, mean ± SD	19.8 ± 14.1	15.0 ± 10.6	0.058
T1SA, mean ± SD	28.1 ± 8.1	25.4 ± 7.9	0.088
C-SVA, mean ± SD	12.7 ± 10.2	18.1 ± 11.6	0.004

Student’s *t*-test.

**Table 2:** Demographic, sport practice, and clinical characteristics of the study population.

Variable	Description
	(N = 64)
Age (years)	
Mean ± SD	24.1 ± 3.4
Median (min.; max.)	24 (18; 35)
Sex	
Female	32 (50)
Male	32 (50)
Practice time	
Less than 3 years	12 (18.8)
3 years or more	52 (81.3)
Position	
Forward	39 (60.9)
Back	25 (39.1)
Neurological history	
No	45 (70.3)
Yes	19 (29.7)
Degeneration	
No	51 (79.7)
Yes	13 (20.3)

**Table 3:** Radiographic parameters and their associations with demographic and clinical variables.

Variable	CL	*p*	T1SA	*p*	C-SVA	*p*	T1SA–CL	*p*
Age (years), mean ± SD	24.0 ± 2.1	0.940	22.3 ± 2.1	0.366	24.3 ± 1.0	0.806	24.6 ± 3.7	0.517
Sex, *n* (%)								
Female	2 (28.6)	0.426	3 (100)	0.238	0 (0)	0.113	10 (66.7)	
Male	5 (71.4)		0 (0)		4 (100)		5 (33.3)	
Practice time ≥ 3 years, *n* (%)	7 (100)	0.331	2 (66.7)	0.470	4 (100)	>0.999	12 (80)	>0.999
Position, *n* (%)								
Forward	6 (85.7)	0.231	0 (0)	0.055	4 (100)	0.149	11 (73.3)	
Back	1 (14.3)		3 (100)		0 (0)		4 (26.7)	
Neurological history, *n* (%)	2 (28.6)	>0.999	1 (33.3)	>0.999	3 (75)	0.075	2 (13.3)	0.195
Degeneration, *n* (%)	0 (0)	0.328	0 (0)	>0.999	0 (0)	0.574	4 (26.7)	0.482
VAS, mean ± SD	2.43 ± 1.62	0.024	1.67 ± 2.08	0.648	3.25 ± 2.75	0.228	1.07 ± 1.34	0.478
IIRP (%), mean ± SD	7.7 ± 7.2	0.072	9.7 ± 7.3	0.067	6.5 ± 4.1	0.409	3.3 ± 3.8	0.342

Comparisons were performed between athletes with normal radiographic parameters and athletes with altered radiographic parameters. Categorical variables were analyzed using Fisher’s exact test, and continuous variables were analyzed using Student’s independent samples *t*-test. CL = cervical lordosis; T1SA = T1 slope angle; C-SVA = cervical sagittal vertical axis; IIRP = Neck Disability Index score (%).

**Table 4:** VAS scores according to demographic and sport-related characteristics.

VAS	Category	*p*
Age (years), r		
Sex	Female/male	0.189
Mean ± SD	1.06 ± 1.44/1.53 ± 1.39	
Median (min.; max.)	1 (0; 5)/1 (0; 6)	
Practice Time	Less than 3 years/3 years or more	0.728
Mean ± SD	1.17 ± 1.64/1.33 ± 1.38	
Median (min.; max.)	1 (0; 5)/1 (0; 6)	
Position	Forward/back	0.007
Mean ± SD	1.64 ± 1.58/0.76 ± 0.93	
Median (min.; max.)	1 (0; 6)/1 (0; 4)	
Neurological history	No/yes	0.755
Mean ± SD	1.33 ± 1.43/1.21 ± 1.44	
Median (min.; max.)	1 (0; 6)/1 (0; 5)	
Degeneration	No/yes	0.056
Mean ± SD	1.41 ± 1.54/0.85 ± 0.69	
Median (min.; max.)	1 (0; 6)/1 (0; 2)	

Student’s *t*-test; r: Pearson correlation.

**Table 5:** Multiple linear regression analyses for VAS and NDI outcomes.

Variable	Factor	Coefficient	Standard Error	t-Value	*p*	R^2^
VAS	Intercept	0.741	0.298	2.489	0.016	0.169
	Sex (male)	0.267	0.361	0.741	0.462	
	Position (forward)	0.703	0.362	1.941	0.057	
	Degeneration	−0.513	0.434	−1.181	0.242	
	CL change	0.894	0.56	1.595	0.116	
IIRP (%)	Intercept	−1.278	4.396	−0.291	0.772	0.191
	Age (years)	0.243	0.179	1.362	0.178	
	Sex (male)	−2.571	1.216	−2.115	0.039	
	Neurological history	2.246	1.329	1.69	0.096	
	CL change	4.351	1.924	2.262	0.027	

Multiple linear regression.

## Data Availability

The original data presented in this study are available from the corresponding author upon reasonable request.
